# The voltage-gated sodium channel Na_v_1.7 associated with endometrial cancer

**DOI:** 10.7150/jca.31544

**Published:** 2019-08-27

**Authors:** Junxiu Liu, Hao Tan, Wancai Yang, Shuzhong Yao, Liang Hong

**Affiliations:** 1 Department of Obstetrics and Gynecology, the First Affiliated Hospital, Sun Yat-Sen University, Guangzhou, China.; 2 Institute of Precision Medicine, Jining Medical University, Jining, China.

**Keywords:** Endometrial Cancer, Na_v_1.7, Voltage-Gated Sodium Channel, Ion Channels.

## Abstract

**Background**: Endometrial cancer is the most common gynecologic malignancy in women in the developed countries. Despite recent progress in functional characterization of voltage-gated sodium channel (Na_v_) in multiple cancers, very little was known about the expression of Na_v_ in human endometrial cancer. The present study sought to determine the role of Na_v_ and molecular nature of this channel in the endometrial cancer.

**Methods**: PCR approach was introduced to determine expression level of Na_v_ subunits in endometrial cancer specimens. Pharmacological agents were used to investigate Na_v_ function in endometrial cancer cells. Flow cytometry were used to test cancer apoptosis, and invasion assays were applied to test tumor metastasis.

**Results**: Transcriptional levels of the all Na_v_ α and β subunits were determined by real time-PCR in endometrial cancer with pair tissues of carcinoma and adjacent nonneoplastic tissue, Na_v_1.7 was the most highly expressed Na_v_ subtype in endometrial cancer tissues. Na_v_1.7 level was closely associated with tumor size, local lymph node metastasis, and 5-year and 10-year survival ratio. Inhibition of this channel by Na_v_1.7 blocker PF-05089771, promoted cancer apoptosis and attenuated cancer cell invasion.

**Conclusion**: These results establish a relationship between voltage-gated sodium channel protein and endometrial cancer, and suggest that Na_v_1.7 is a potential prognostic biomarker and could serve as a novel therapeutic target for endometrial cancer.

## Introduction

Endometrial cancer is a major cause of morbidity for women worldwide, and approximately 3% of women develop endometrial cancer at some point during their lifetimes 1. The 5-year survival rate for women with stage I endometrial cancer is 90%, it drops to 57% in patients with stage III, and to 20% in patients with stage IV 2. As the determinant of survival in endometrial cancer is the stage of disease at diagnosis, the early detection and effective therapy are of considerable importance. Recently, ion channels have emerged as new biomarkers for human cancers, and some have been shown to correlate with the main hallmarks of the cancer process and serve as pharmacological targets in the cancer chemotherapy 3.

The voltage-gated sodium channels (Na_v_s) are responsible for the fast action potentials involved in nerve and cardiac conduction 4, they were recently found to play crucial roles in cancer development and progression 5. The family of sodium channels has nine members named Na_v_1.1 through Na_v_1.9. Among them, the Na_v_1.5 has been shown to be associated with colon cancer and breast cancer metastasis 6, 7; inhibition of Na_v_1.6 reduced invasiveness of cervical cancer primary culture cells 8; and in prostate cancer, Na_v_1.8 expression was revealed to be closely correlated with pathologic stage of cancer specimens 9. Despite recent progress in the functional characterization of sodium channel in multiple cancers, very little was known about the expression of Na_v_ in human endometrial cancer; furthermore, the molecular basis of sodium channel in this type of cancer has not yet been identified.

In this study, we used primary cultures to investigate the potential role of Na_v_ in endometrial cancer. The present study aimed to determine whether voltage-gated sodium channel protein functionally expressed in the endometrial cancer with metastatic potential, whether their expressions are associated with clinical outcome, and what molecular nature of sodium channels are in the endometrial cancer, whether their activities contribute cellular behaviors integral to metastasis.

## Materials and Methods

### Patients and tissue samples

A total of 80 surgical specimens of endometrial cancer tissues were collected from patients at the Department of Obstetrics and Gynecology, the First Affiliated Hospital, Sun Yat-Sen University from 2006 to 2016 without prior radiotherapy or chemotherapy. Twenty paired surgical tumor and normal adjacent tissues were obtained with the patients' consent from the patients registered at the First Affiliated Hospital. The normal adjacent tissue, defined as histologically benign-appearing tissue and judged by an experienced pathologist, is acquired from the margins of the tumor resection. A separate set of frozen tumor specimens for Kaplan-Meier analyses were obtained from sixty patients. The Na_v_1.7 expression determined by quantitative PCR was evaluated in those specimens, *MRPL19* was used as the reference gene to normalize Na_v_1.7 expression, the group Na_v_1.7-High or Na_v_1.7-Low were defined as scores above or below the median. The study was approved by the Institutional Review Board of First Affiliated Hospital of Sun Yat-sen University (Guangzhou, China). Patient studies were conducted in accordance with ethical guideline of Declaration of Helsinki.

### Cell culture

Fresh endometrial cancer biopsies were digested with collagenase (1 mg/ml; Sigma-Aldrich) in Hanks Balanced Salt solution (HBSS) at 37°C for 30 min. The suspended cells were collected by centrifugation at 500 r.p.m for 5 min at 4°C, cells were transferred to a fresh tube containing HBSS, washed and centrifuged again. Then the primary cells were plated on coverslips in Falcon polystyrene microplates 6-well plates, and maintained in Dulbecco's modified Eagle's medium (DMEM, Gibco) with 10% fetal bovine serum (FBS, Gibco), 100 U/ml penicillin and 100U/ml streptomycin (Invitrogen) in a 37°C incubator with 5% CO_2_ 10, 11.

### RNA isolation and cDNA synthesis

Total RNA was extracted from endometrial cancer and nonneoplastic endometrial tissues using TRIzol RNA extraction agent (Invitrogen) according to the manufacturer's instructions 12. Only RNA that resulted in an A260/280 ratio of 1.8-2.0 was reverse transcribed to generate cDNA. Synthesis of cDNA was carried out with SuperScript II RNase Reverse Transcriptase (Invitrogen) and primers (Invitrogen) at 42°C with 2µg of total RNA as template, in a final volume of 20µl. Negative controls for the reverse-transcription reaction were prepared by omitting the RT enzyme 13, 14. For the reverse transcription-PCR, the relative intensity of Na_v_1.7 mRNA expression was measured by densitometry (ImageJ, Bethesda, USA). For conventional end-point PCR, 100 ng of cDNA was amplified following addition to a 30µl mastermix containing dNTPs, Platinum Taq DNA polymerase (Invitrogen) enzyme and appropriate forward and reverse primers for the desired Na_v_ α subunits target gene. Amplicons were visualized under UV light following separation through a 1% agarose gel containing ethidium bromide.

### Quantitative real time-PCR

Quantitation of Na_v_ α and β subunits and *MRPL19* mRNA was carried out by real time PCR using SYBR I green chemistry on an MJ Chromo 4 thermal cycler (BioRad, USA) 15-17. Approximately 1 ng/µl of cDNA was added to Platinum SYBR Green qPCR Supermix-UDG (Invitrogen) and primers in a 25µl reaction. Standard curves were generated from serially diluted endometrial cDNA and the Na_v_ subunits transcripts quantitated by normalizing expression relative to the reference gene *MRPL19*. The CT (threshold cycle) value was determined in each experimental group. The data normalization was performed by using the CT value from human MRPL19 (*ΔCT=CT_Nav subunit_ -CT_MRPL19_*), the *ΔCT* for EC samples was then normalized to NE samples (*ΔΔCT=ΔCT_EC_-ΔCT_NE_*), the *ΔΔCT* was converted to -2*^-ΔΔCT^* to calculate the relative expression levels of Na_v_ α and β subunits 8. The primer pairs used for polymerase chain reaction for all Na_v_ α and β subunits and *MRPL19* were shown in the Table 1.

### Flow cytometry analysis

The annexin V-fluorescence isothiocyanate (FITC)/PI apoptosis detection kit (BD Biosciences) was used to assess apoptosis. After 48 hours' drug incubation, the cells from each sample (1×10^5^) were re-suspended in 200μl of staining buffer and mixed with 10μl of annexin V-FITC for 15 min. After adding 200μl staining buffer and 10μl PI, flow cytometry was performed to analyze the percentage of apoptotic cells 18, 19.

### Invasion assays

The endometrial cancer cells (1×10^5^) were seeded in the top well of a Matrigel-coated invasion chamber (BD Biosciences) in DMEM containing 0.1% FBS with or without pharmacological agents (Tetrodotoxin, veratridine or PF-05089771). The bottom well was filled with 750μl DMEM containing 10% FBS as a chemoattractant. After 6-48 hour, non-invading cells were scraped from the upper side of the insert using a cotton swab. Invading cells on the bottom of the insert were fixed and stained with Diff-Quick Stain (IMEB Inc., USA) according to manufacturer's instructions 20-22. The total number of invading cells was counted for each insert under a light microscope (Nikon Corporation, Japan).

### Data analysis

All data are presented as the means ± standard error of the mean. The n value denotes the number of independent experiments conducted. Significance between means was determined using either the two-tailed Student's paired t-test or one-way analysis of variance with Dunnett's multiple comparisons test. Kaplan-Meier and log rank tests were used to assess differences in overall survival or disease-specific survival by Na_v_1.7-High *vs* Na_v_1.7-Low. *P*<0.05 was considered to indicate a statistically significant difference.

## Results

### Expression levels of Na_v_ α subunits in endometrial cancer and nonneoplastic endometrial samples

**Fig 1:**
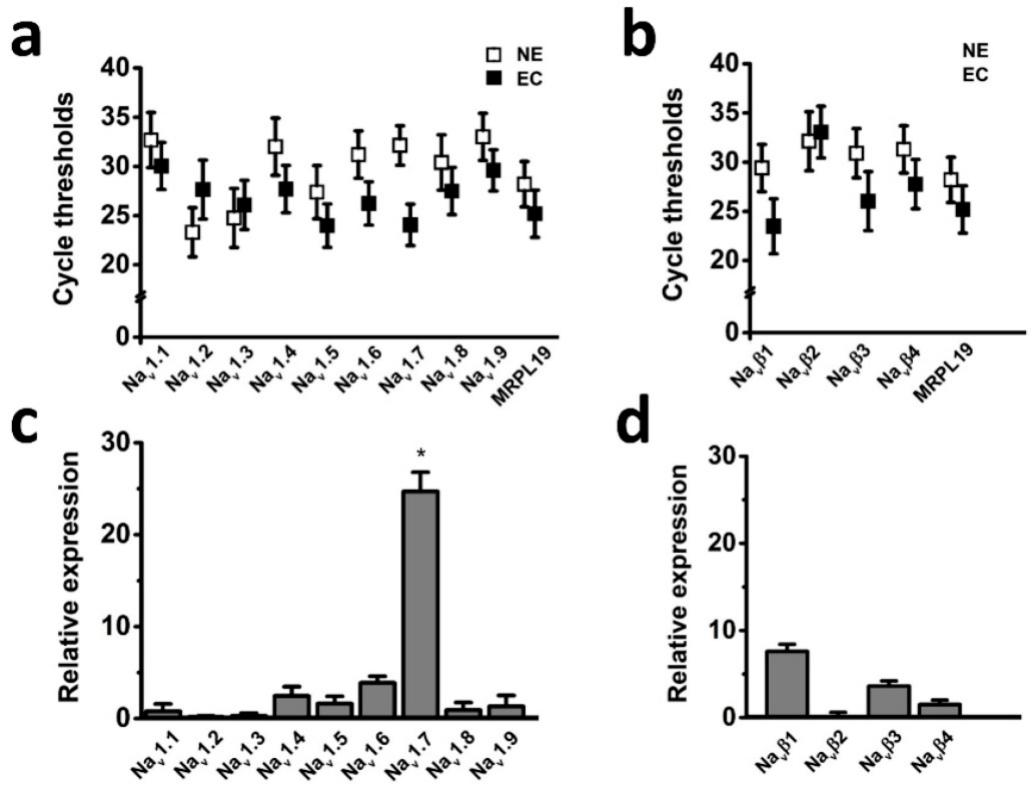
Expression levels of Na_v_ subunits in endometrial cancer with pair tissues of carcinoma (EC) and adjacent nonneoplastic tissue (NE). **a-b** Mean cycle threshold value of Na_v_ α subunits (**a**), Na_v_ β subunits (**b**), and housekeeping gene *MRPL19* in primary EC biopsies (black boxes, n=6) and NE samples (white boxes, n=6). **c**-**d** Real-time quantitative PCR of Na_v_ α (**c**) and β subunits (**d**) mRNA levels fold changes in EC and NE samples, bars showed the average fold-change ratios of Na_v_ α or β subunits gene expression levels between EC and NE tissues for the indicated Na_v_ subunits. *MRPL19* was used as the reference gene to normalize Na_v_ subunits gene expression (n=6, *P<0.05).

The voltage-gated sodium channel (Na_v_) has been shown to play important roles in cancer development and progression 5; however, it has not been known whether Na_v_ expression level has relationships with endometrial tumor malignancy. To test this possibility, we examined the mRNA expression level of Na_v_ in endometrial cancer specimens. Transcriptional levels of the all Na_v_ α and β subunits were determined by real time-PCR in the six cases of endometrial cancer with pair tissues of carcinoma (EC) and adjacent nonneoplastic tissue (NE), the mitochondrial ribosomal protein L19 (*MRPL19*) was introduced as the reference gene to normalize Na_v_ subtype gene expression 23. The cycle threshold values were plotted to compare gene expression, and the real time-PCR analyses revealed that Na_v_1.7 was the most highly expressed Na_v_ subtype in the tissues (Fig. 1), and relative mRNA expression of Na_v_1.7 in EC biopsies were approximately 25-fold higher than in NE samples (Fig. 1c), indicating that overexpression of Na_v_1.7 was associated with endometrial tumorigenesis.

### Na_v_1.7 expression was associated with endometrial cancer metastasis and clinical outcome

**Fig 2:**
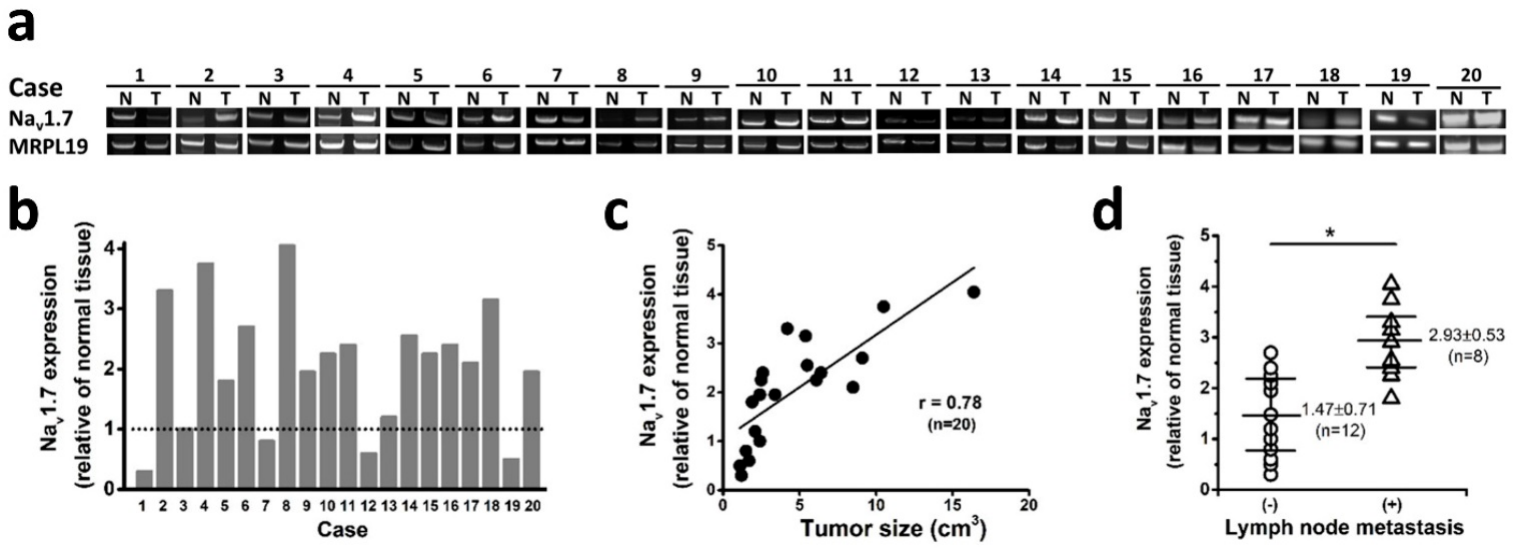
Na_v_1.7 expressions associated with endometrial cancer metastasis and clinical outcome.** a** Na_v_1.7 expressions were determined in EC and NE tissues from 20 endometrial cancer patients. N and T, nonneoplastic endometrial tissues, and tumor areas of the same endometrial cancer patients, respectively. **b** Na_v_1.7 expression level in tumor biopsies. Nav1.7 level in adjacent normal tissues were used as control, housekeeping gene *MRPL19* was used as the reference gene to normalize Na_v_1.7 expressions. **c** The association between Na_v_1.7 expression level and tumor size in the same surgical biopsies of endometrial cancer patients (r=0.78, n=20, *P*<0.05). **d** The tumor expression level of Na_v_1.7 was higher in the patients with local lymph node metastasis (2.93 ± 0.53, n=8) than those without lymph node metastasis (1.47 ± 0.71, n=12, ^*^P<0.05).

To investigate if Na_v_1.7 expression has clinical significance in tumor progression in endometrial cancer, we analyzed 20 sets of endometrial cancers with pair tissues of EC and adjacent NE tissue, and found that 75% cases (15 of 20 endometrial cancer) expressed significantly elevated level of Na_v_1.7 expression compared with paired adjacent normal tissue (Fig. 2a-b). Nav1.7 expression was downregulated in 4 cases, one possible reason is due to heterogeneity or individual difference in patients. More importantly, the Na_v_1.7 expression level was closely correlated with tumor size (Fig. 2c), a crucial indicator for the state of disease progression in human endometrial cancer 24. In addition, the level of Na_v_1.7 expression in tumor tissues was significantly higher in the group of local lymph node metastasis (Fig. 2d).

**Fig 3:**
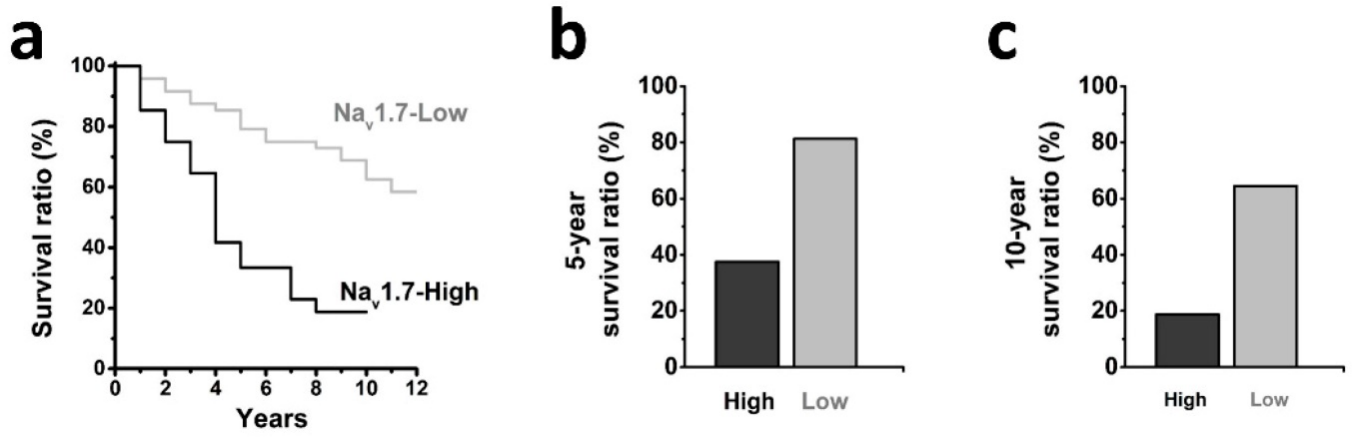
** a** Kaplan-Meier analyses showing the correlation between the levels of Na_v_1.7 and the overall survival of patients with endometrial cancer (Na_v_1.7-High, n=38; Na_v_1.7-Low, n=21; *P*<0.05, log-rank test). **b-c** High Na_v_1.7 expression correlated with decreased survival in endometrial cancer, the 5-year survival ration (**b**) was decreased in Na_v_1.7-High group (38%) compared with Na_v_1.7-Low group (81%), and the 10-year survival ration (**c**) was decreased in Na_v_1.7-High group (19%) compared with Na_v_1.7-Low group (62%).

We further determined the association between tumor expression of Na_v_1.7 and clinical outcome of patients with endometrial cancer (Fig. 3a), and observed that patients with high-level tumor expression of Na_v_1.7 exhibited a shorter 5-year and 10-year survival ratio as compared with the Na_v_1.7-low group (38% *vs* 81% and 19% *vs* 62%, respectively) (Fig. 3b-c).

### Nav1.7 involved in endometrial cancer apoptosis

We next asked whether Na_v_1.7 activities contribute to the development of endometrial cancer, we tested effects of veratridine and PF-05089771 on endometrial cancer cells. Veratridine is a Na_v_1.7 activator 25, it was able to induce persistent Nav1.7 currents 26, and inhibited channel inactivation and generated enhanced window currents. PF-05089771 was previously identified as a state-dependent Na_v_1.7 specific inhibitor interacting with Na_v_1.7 voltage-sensor domain of domain IV 27, 28. We used flow cytometry analysis to investigate the consequences of veratridine and PF-05089771 on the cancer cell apoptosis. The cells were divided into three groups as shown in Suppl. Fig. 1a-c. The results showed that PF-05089771 were able to increase the number of early and late apoptotic cells, whereas veratridine reduced late apoptosis (Suppl. Fig. 1d), indicating that the Na_v_1.7 may have a critical role in endometrial cancer development.

### Endometrial cancer invasion is mediated by Na_v_1.7

**Fig 4:**
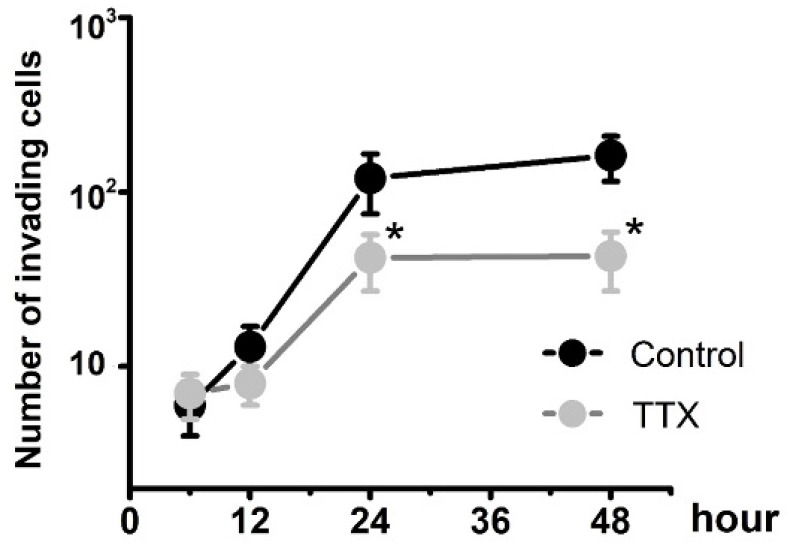
Endometrial cancer invasion is mediated by voltage-gated sodium channel**.** Total number of invading cancer cells was in the absence (Control) or presence of 10 µM TTX over the time period (from 6 to 48 hours). Data were from 6 independent experiments in each group, shown were means ± SEM. ^*^P<0.05 versus Control.

**Fig 5:**
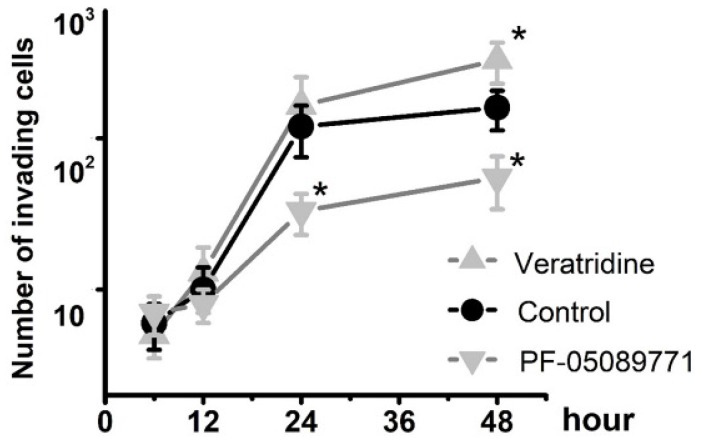
Total number of invading cells was increased in the presence of 100µM veratridine and attenuated in the presence of 100µM PF-05089771 over the time period. Data were from 6 independent experiments in each group, shown were means ± SEM. ^*^P<0.05 versus Control.

To determine whether Na_v_1.7 sodium channel participates in metastatic cell behaviors, the invasion assays were performed with endometrial cancer cells. The role of sodium channel in EC cells was assessed using the specific blocker Tetrodotoxin (TTX). The results revealed that TTX attenuated the relative invasiveness of EC cells. As shown in Fig. 4, the treatment with 10 µM TTX for 24 hours significantly decreased the number of invading cells. We then introduced veratridine and PF-05089771 to test roles of Na_v_1.7 in EC. The results revealed that 100µM veratridine increased invasion over 48-hour time period compared to control (Fig. 5). On the contrary, PF-05089771 significantly attenuated the relative invasiveness of EC cells, treatment with 100µM PF-05089771 remarkably reduced number of invading cells (Fig. 5). These results, together with the data that patients with local lymph node metastasis have higher level of Na_v_1.7 expressions (Fig. 2d), showed that the Na_v_1.7 plays a critical role in EC metastatic behaviors. And the effects of veratridine on cancer cell invasion were due to enhancement of Na_v_1.7 activities, whereas blockade of Na_v_1.7 by PF-05089771, attenuated endometrial cancer cell invasion.

## Discussion

This is the first study revealing the connection between voltage-gated sodium channels and endometrial cancer, examining the role of Na_v_1.7 sodium channel in this type of cancer.

Ion channels were well known to play significant roles in the growth and migration of cancer cells and contribute to multiple aspects and stages of cancer progress 3. There were several ion channels implicated in endometrial cancer. The hERG K^+^ channels were found to be expressed with a higher frequency in primary human endometrial cancer compared to non-cancerous tissues 29. The Ca^2+^ channel Ca_v_1.3 required for estrogen-stimulated Ca^2+^ influx contributed broadly to the development of endometrial cancer 30. A recent study reported that volume-activated Cl^-^ channel play roles in endometrial tumor invasion and migration 31. Compared to Ca^2+^, K^+^, and Cl^-^ channel, however, the role of voltage-gated Na^+^ channel in the endometrial cancer remains unknown.

The voltage-gated Na^+^ channel has been established to be associated with metastatic cell behavior in cancer 5, 32, 33, several Na^+^ channel isoforms were identified to be expressed in different cancers, these included Na_v_1.5 in breast and colon cancers 6, 7, Na_v_1.6 in cervical cancer 8, and Na_v_1.8 in prostate cancer 9. In this study, we characterized Na_v_ isoform in human endometrial cancer. We used real-time-PCR to determine transcriptional levels of sodium channel α and β subunits, and discovered that Na_v_1.7 α subunit in EC samples were around 25-fold higher than in NE biopsies, Na_v_1.7 overexpression in tumor tissue was noted in 75% cases of endometrial cancer. More importantly, the level of Na_v_1.7 expression was significantly associated with tumor size and survival in tumor tissues. We showed that Na_v_1.7 associated with endometrial cancer development, the Na_v_1.7 activator veratridine reduced endometrial tumor cell apoptosis and promoted cancer invasion, and inhibition of Na_v_1.7 by PF-05089771 increase the number of apoptotic cells and attenuated invasive potential of cancer cells.

There are several theories regarding how Na_v_ contribute to tumor progression. One explanation is that function upregulation of Na_v_, consequently activate the Na^+^/H^+^ exchanger (NHE) and enhance H^+^ efflux, thus leading to increased intracellular alkalinisation and decreased extracellular pH. In cancer cells, increased glycolytic metabolism gives rise to an excessive production of intracellular acidity; as a result, intracellular alkalinisation potentially facilitates cancer metabolism 34. Another theory proposed that Na_v_ could activate Na^+^/Ca^2+^ exchanger (NCX), leading to the entry of Ca^2+^ through the NCX, which induces Ca^2+^-dependent signaling to promote cancer cell proliferation and metastasis 35, 36. In this study, Na_v_1.7 activator veratridine and inhibitor PF-05089771 affected endometrial cancer apoptosis and invasion, indicating that Na_v_1.7 have crucial roles for endometrial cancer progression. However, whether Na_v_1.7 activated NHE to increase H^+^ efflux to provide a favorable environment for endometrial tumor invasion, or induced Ca^2+^-dependent signaling by stimulating NCX activity to accelerate development of endometrial cancer, remain unclear; further studies are required to address these uncertainties.

In summary, the present study established a relationship between voltage-gated sodium channel protein and endometrial cancer. The Na_v_1.7, functionally expressed in the endometrial cancer, has strong links with clinical outcome. Its activity significantly contributes endometrial tumor progression. These findings highlight the importance of Na_v_1.7 in cancer development, and may provide novel insights into early detection and chemotherapeutics for endometrial cancer.

## Supplementary Material

Supplementary figure.

## Figures and Tables

**Table 1:** Primers used in PCR determining expression of Na_v_ subunits in endometrial cancer

Protein	Gene	GenBank accession	Forward primer sequence (5´ to 3´)	Reverse primer sequence (5´ to 3´)
Nav1.1	*SCN1A*	NM_001202435.2	CCCGACTGTGACCCTAATAAAG	CAGAGGCTCTGCACTTTCTTC
Nav1.2	*SCN2A*	NM_001040142.1	GTGCTGGTCATTTTCTTGGGC	CTTGATTCAGCAGATGCGGC
Nav1.3	*SCN3A*	NM_006922.3	GGCAAAGGGAAGATCTGGTGG	CCATAAGCAACCCATTTGAGAAGC
Nav1.4	*SCN4A*	NM_000334.4	CTCGAGCTGGACCACCTTAAC	CGGACGAGTTCCCATCATAG
Nav1.5	*SCN5A*	NM_198056.2	CTTGGCCAAGATCAACCTGCTC	GATGACTCGGAAGAGCGTCG
Nav1.6	*SCN8A*	NM_014191.3	GCAGCCGGGAAAACATACATG	GCCTGTGCCTCTTCCTGTTGC
Nav1.7	*SCN9A*	NM_001365536.1	GCAAGGCGAAGCAGCAGAAC	GGCTTGGCTGATGTTACTGCTG
Nav1.8	*SCN10A*	NM_006514.3	CCTCTCTCCACTCCCACAATG	CACACTGCCATGACTAGCCC
Nav1.9	*SCN11A*	NM_014139.2	CTGACTGTGGTCCTGGTCATTG	CGATCCATTCCCCGCAGAGG
Navβ1	*SCN1B*	BC112922.1	GAGACCACCGCCGAGACCTTC	CGCCAGAGTGGTTGTAGGTG
Navβ2	*SCN2B*	AY358945.1	GTTCCTCCAGTTCCGCATGAAG	GACCTGCAGATGGATCTTGCC
Navβ3	*SCN3B*	CH471065.1	GGGTCAGTGTCTGCTTCCCTG	CCTCCTGGTGGCCATTCCG
Navβ4	*SCN4B*	AY149967.1	GGACCTGGAGTTCAGCGAC	GCAGGATGAGGATGAGGAG
MRPL19	*MRPL19*	NM_014763.3	GCCAGTGGAAAAATCAGCCAG	GAATCTCCTGGACCCGAGG

## Abbreviations

Na_v_: voltage-gated sodium channel
Na_v_1.7: voltage-gated sodium channel α-subunit encoded by the *SCN9A* gene
EC: endometrial cancer tissues
NE: nonneoplastic endometrial samples
TTX: Tetrodotoxin
