# Supplementary material for: The voltage-gated sodium channel Nav1.7 associated with endometrial cancer
Source: J Cancer. 2019 Aug 27;10(20):4954–60. doi: 10.7150/jca.31544 (PMC6775510; doi:10.7150/jca.31544)
Supplement: Supplementary file 1 — Supplementary figure. [file jcav10p4954s1.pdf]

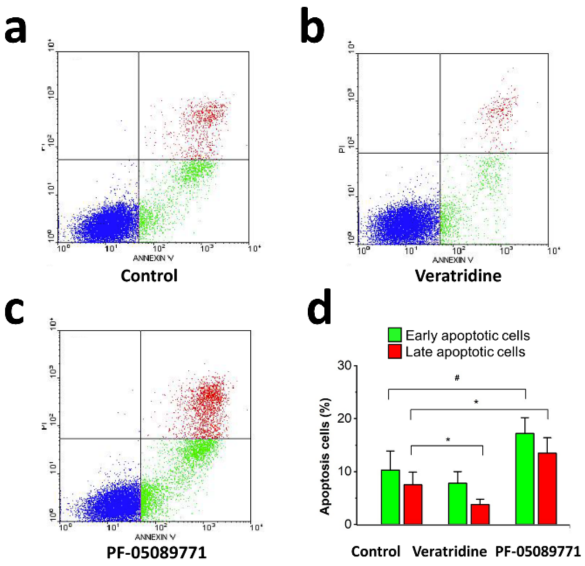

Supplementary Fig.1 Nav1.7 involved in endometrial cancer apoptosis. **a-c** Endometrial cancer cells collected and analyzed for apoptosis by flow cytometry in various groups: Control (**a**), cells treated with 100μM veratridine (**b**) or 100μM PF-05089771 (**c**). Early apoptotic cells (low right quadrant, green), late apoptotic cells (upper right quadrant, red) and viable cells (low left quadrant, blue) were determined using double labeling annexin V and PI. **d** PF-05089771 enhances EC cells early and late apoptosis, veratridine reduces EC cells late apoptosis. n=7 for each group; data shown are means ± SEM. $^{\#}P<0.05$ versus Control early apoptotic cells. $^{*}P<0.05$ versus Control late apoptotic cells.
